# Environmental degradation, climate change and health from the perspective of Brazilian Indigenous stakeholders: a qualitative study

**DOI:** 10.1136/bmjopen-2023-083624

**Published:** 2024-09-24

**Authors:** Antonio José Grande, Ieda M A V Dias, Paulo T C Jardim, Alessandra Aparecida Vieira Machado, Jacks Soratto, Maria Inês da Rosa, Luciane Bisognin Ceretta, Xanthi Zourntos, Regeane Oliveira Suares, Seeromanie Harding

**Affiliations:** 1 Universidade Estadual do Mato Grosso do Sul, Dourados, Brazil; 2 Federal University of Rio Grande do Sul, Porto Alegre, Rio Grande do Sul, Brazil; 3 Universidade do Extremo Sul Catarinense, Criciuma, Brazil; 4 Department of Population Health Sciences, King's College London, London, UK

**Keywords:** mental health, community-based participatory research, statistics & research methods

## Abstract

**Abstract:**

**Background:**

The WHO identifies climate change as the most significant threat to global health systems. Indigenous peoples, whose lives are deeply intertwined with nature, are particularly vulnerable to the impacts of these changes.

**Objective:**

This study aimed to understand the perspectives of Indigenous stakeholders and public services managers on the interconnectedness of climate change and Indigenous health.

**Design:**

A qualitative study with 22 Indigenous stakeholders and public service managers on climate change and perceived impact on Indigenous health.

**Setting and participants:**

Indigenous stakeholders and public service managers on climate change and perceived impact on Indigenous health from Brazil. Data was collected through interviews incorporating two vignette videos depicting environmental and health scenarios. Thematic content analysis was used to analyse the data.

**Results:**

The analytical process yielded six subcategories that were further grouped into three overarching thematic macro-categories: environmental degradation and climate change in the context of Indigenous peoples; environment, vulnerability and impact on Indigenous mental health; and actions and public health policies for Indigenous peoples.

**Conclusion:**

The perspectives of Indigenous stakeholders and public service managers on the interconnectedness of climate change and Indigenous health were deeply entrenched in their lived experiences of loss of their lands from deforestation and environmental degradation. They argued strongly for the strengthening of public health policies aimed at the Indigenous peoples, to face many challenges, especially suicide, and to have a voice in decision-making. A sensitive approach that values Indigenous peoples' connections with nature is fundamental to promote their health and well-being.

**STRENGTHS AND LIMITATIONS OF THIS STUDY**

- A blend of culturally and environmentally focused vignettes and interviews.
- Inclusion of indigenous voices in shaping policy and programme development.
- Narrated interviews that offer community perspectives.
- Underrepresentation of various indigenous ethnicities.
- Small sample size leading to less precise measurements.

## Introduction

 Brazil’s Indigenous population, currently comprising over 1.65 million according to the 2022 Census, has seen a notable increase due to birth rates, which are higher than that of the non-Indigenous population,^1^ and to an increase in self-recognition of Indigeneity as a result of political participation of Indigenous peoples.^2^ As with Indigenous peoples globally, compared with non-Indigenous population in Brazil, the Indigenous population experiences poorer health and well-being.^3^ A recent report on mortality trends in 2000–2016 showed increasing mortality for both females and males for most age groups.^4^ The highest increases were observed for those aged≥60 years and 10 to 19 years. In children aged <5 years, the main causes of death were infectious and parasitic diseases, as well as respiratory diseases.^5^ Between 5-59 years, external causes ranked first and were responsible for more than half of all deaths among those aged 10–19 years.^3^ Circulatory diseases were the most common cause of deaths for those aged ≥60 years.^4^,^6^ These trends are linked to poor provision of basic social and health services including precarious sanitation conditions that make children vulnerable to infections, conflicts between farmers and Indigenous people, urban violence, and a general erosion of Indigenous traditions and customs in agriculture, hunting and fishing.^7^

Environmental degradation poses a critical threat to Indigenous health. Ecosystem degradation, rising temperatures and extreme weather events are major concerns, with the WHO identifies climate change as the most significant threat to global health systems. Indigenous peoples, whose lives are deeply intertwined with nature, are particularly vulnerable to these changes.^8^,^10^ A recent study found high levels of mercury in hair samples and mouth swabs among members of the Yanomami Indigenous group living in nine villages in the upper Mucajai river in the northern state of Roraima where illegal gold mining is common.^6^ Mercury consumption was high due to contaminated fish, which is one of the Yanomami’s main food sources. Cognitive deficits among children were observed in half of the children surveyed in nine villages. There are also concerns that deforestation and degradation are linked to high rates of substance use and suicides among Brazil’s Indigenous communities.^7^ Indigenous knowledge has been used for centuries for local adaptations for environmental sustainability. Indigenous scholars have long argued that their knowledge is critical for long-term sustainable solutions for biodiversity loss, water scarcity, pollution, sustainable livelihoods and general environmental resilience.^8 9^

The Indigenous participants in this study are from South and Midwest Brazil. They strive to maintain their demarcated lands and spiritual connection to nature, and possess a rich storytelling tradition that reinforces their identity and belonging.^8^ Their schools promote the retention of Indigenous knowledge and practices that can resolve local environmental challenges.^10^ For example, the Indigenous schools integrate growing and eating of cultural foods into the curriculum, oral learning methods and participation in village activities with community leaders, shamans, parents, and elders.^11 12^

This study aimed to understand the perspectives of Indigenous stakeholders and public service managers on the interconnectedness of climate change and Indigenous health.

## Methods

### Theoretic framework

This qualitative study employed in-depth interviews to gather and analyse perspectives on the impacts of climate change on Indigenous health. Grounded in the principles of political ecology, which examines conflicts and socio-environmental changes, along with their interactions and relations with human societies, this theoretical lens guided both the study design and the subsequent development of analytical categories.^13 14^ Methodological rigour was ensured through adherence to the Consolidated Criteria for Qualitative Research Reports.^15^

### Trusting relationships

We collaborated with key Indigenous stakeholders in Mato Grosso do Sul, Brazil. Building on long-established community-academic partnerships, our research team prioritised cultural integrity and agency by integrating Indigenous knowledge and values throughout the research process. The research question emerged organically from our ongoing collaborative work with this community since 2017.

### Setting

While the study was based in Campo Grande, Mato Grosso do Sul, the snowball sampling method resulted in data collection across six cities (Brasilia, Campo Grande, Porto Alegre, Guarita, Dourados and Terenos) and three Brazilian states (Mato Grosso do Sul, Federal District of Brasilia and Rio Grande do Sul).

### Participant selection

The recruitment of participants took place through the announcement on social networks and e-mail contacts of the authors. The participants voluntarily agreed to participate in the research and were recruited using snowballing.^16^ In this non-probabilistic sampling technique, the individual selected intentionally to participate in the study invites or indicates new participants from their social or professional network. Of an initial 30 interested participants, 22 participants met the inclusion criteria (see section on ‘Participants’). One of the participants indicated a new contact who, in turn, referred others, and so on, until the 22nd participant, by which time data saturation had been reached.^17^

### Participants

The study involved 22 Indigenous stakeholders/public service managers. Inclusion criteria were as follows: (a) public service managers: individuals over 18 years old, holding management roles across municipal, state or federal levels in executive, legislative or judicial branches and operating in Indigenous areas; (b) Indigenous peoples: individuals aged over 18 years old, self-identifying and recognised as belonging to an Indigenous ethnic group with distinct cultural characteristics from the national society. The characterisation of the research participants is presented in table 1.

**Table 1:** Characterisation of research participants

Variables	N	%
Gender		
Female	12	54,5
Male	10	45,5
Age group		
20–29 years	5	22,7
30–39 years	8	36,4
40–49 years	7	31,8
50–59 years	2	9,1
Training		
Technical	2	9,1
Undergraduate	5	22,7
Post-graduate	15	68,2
Working time in Indigenous health/public service management		
Less than 1 year	1	4,5
Between 1 and 5 years	5	22,7
Between 6 and 10 years	7	31,9
Between 11 and 20 years	8	36,4
More than 20 years	1	4,5
I have another job		
No	17	77,3
Yes	5	22.7

Just over half of the participants were female, and two-thirds were between 30–49 years old and had a post-graduate qualification. Two-thirds had spent between 6–20 years working in Indigenous health or public service management from Terena and Kaingang ethnicity.

### Data collection

The data collection process was developed by a university-employed researcher, who was trained in qualitative methods. The interviewer worked in the same municipality as the data collection site, but in different services.

The data collection was carried out between August 2021 and April 2022, using vignettes and semi-structured interviews. The interviews lasted approximately 60 min and were carried out according to the availability of participants, using remote communication technology with audio and video recording, for later transcription. The aim of the interview was for the interviewee to discuss the proposed topic, without losing sight of the research objectives, following a script or previously elaborated structure.^15^

The interviews initially answered questions relating to their gender, age, length of time working with Indigenous health/management and whether they have another job. The interviews were conducted online, and are available as a online supplemental file. During the interviews, two vignettes in the form of videos were presented to the participants. Vignettes are known to (a) enhance realism by considering various contextual factors and guiding participants' focus towards specific aspects of the research question, (b) offer a standardised stimulus and improve reliability, and (c) reduce social desirability bias and strengthen participant engagement.^15^ The first video illustrates a Brazilian Indigenous activist who participated in the official opening of the Climate Summit Conference (COP26) held in Glasgow in Scotland in 2021 and who spoke about Indigenous people and their important role in practices that are helping to mitigate or adapt to climate change: https://bit.ly/3SwZsmq..

The second video shows mental health problems and access to health services for Indigenous people that narrates a case that occurred with a child is available at https://bit.ly/3A6g6Tt..

To support the research, we used the tools made available by the G-Suite package from Google. The interviews were carried out virtually via Google Meet. The signing of the consent term and the completion of the profile questions will be answered via Google Forms before the start of the interview. The recording of the interview and transcripts are stored on Google drive. Finally, the collaborative documents for transcription and analysis were recorded in Google documents.

A summary of the device functionality and objective is described in table 2.

**Table 2:** Functionalities and objectives for the use of the G-Suite tools

Device	Functionality	Objective
Google Meet	Realisation of synchronous interactions of audio, video, texts and projection of electronic content.	Perform interviews
Google Forms	Access and submission of the free and informed consent form.	Completion of questionnaires and signature of the informed consent form
Google Drive	Storage of electronic documents of various formats.	Storage of the recordings of interviews and two transcription documents and data analysis.
Google Documents	Collaborative creation and editing of text documents by two researchers.	Transcription of the recorded treatment, documentation and analysis of two data

### Data analysis

Data analysis was conducted using thematic content analysis, a method that involved dissecting the text into units to uncover the underlying nuclei of meaning within the communication. These nuclei were then regrouped into distinct classes or categories.^18^ For data analysis, to reach the manifest and latent meanings in the material, Content Analysis was carried out using the Atlas.ti Software, version 9, according to analytical precepts aimed at health,^19^ and was divided into three stages:

1. Pre-analysis: carried out through transcription, reading, correction of language errors and prior organisation of all interviews. After this organisation, the text documents were uploaded into the software.
2. Exploration of the material: this stage consisted of the process of immersion in the subjects, from the selection of expressive excerpts converging to the research objective, creation of codes and groupings into converging themes.
3. Data processing: the moment of inference consisted of two authors relating excerpts from the transcriptions, codes and groupings of codes to form subcategories and categories. Rounds of evaluation were carried out by the study authors to establish consensus on the codes that would be used in each grouping. The citation excerpts from the chosen codes were those that had greater frequencies, co-occurrence, that is, connections with other codes, and were more significant from the researcher’s perspective, in order to more clearly represent the theme covered. The initial results were tabulated to show the network of words that emphasised the most expressive concepts in two selected passages. Excerpts of quotes were then selected to bring together a coherent narrative across the interviews.

### Ethics

The research project was approved by the Research Ethics Committee of the University of Brasília (CEP/UNB) and by the National Research Ethics Commission (CONEP) under opinion no. 4,279,173, also respecting ethical standards established in Resolution CNS No. 510 of 2016 and Resolution CNS No. 466 of 2012 of CONEP (CAAE: 37321520.4.0000.5020).

The anonymity of the participants was preserved by the inclusion of an alphanumeric code composed of letter P for participants followed by a cardinal number. For example: P1 refers to participant number one and so on.

The recording audios were deleted after transcription, and the transcriptions were saved on the external hard drive held by the study coordinator.

### Patient and public involvement

The study design was co-developed in close collaboration with Indigenous community members and leaders, building on established relationships from previous studies. This participatory approach ensured that the research questions, methodologies and data collection methods were culturally relevant, respectful and responsive to the unique needs and priorities identified by Indigenous communities. Upon publication, study results will be shared directly with all participants through culturally appropriate channels, including face-to-face meetings for local participants and online platforms for those residing further away, fostering an ongoing dialogue and knowledge exchange.

## Results

The analytical process allowed the selection of 1101 citation excerpts, 21 codes and six subcategories grouped into three thematic macrocategories is presented in table 3.

**Table 3:** The number of quoted passages according to codes, subcategories and thematic categories on climate change and health in the Brazilian Indigenous context

Categories and subcategories	Codes
Environmental degradation and climate change in the context of Indigenous peoples	
Public policies	Indigenous adolescents
	Demarcation of unworthy lands
	Water
	Joint intersectoral actions
Environment	Climate change and environmental degradation
	Environmental preservation
	Forest use
	Agriculture
Environment, vulnerability and Indigenous mental health	
Problems of Indigenous peoples	Culture
	Lack of access
	Difficulty integrating into society
Mental health	Suicide
	Experiences
	Sense of belonging
	Drugs and alcohol
Public health actions and policies for Indigenous peoples	
	Immediate actions
	Indigenous voices
	They haven't seen reality
Suggestions	Community
	Nature
	Dignity and health

The category that commonly identified was environmental degradation and climate change in the context of Indigenous peoples, reinforcing the need for public policies that relate to Indigenous adolescents and the impacts of climate changes and environmental degradation. This intrinsic connection to the environment makes the integration of the knowledge and experiences of Indigenous communities essential for environmental preservation. The following quotes represent this analytical category:

…We Indigenous People do not understand this land and territory as something to be explored, as something to be devastated, as something to serve us, an idea that the environment is at the service of human beings. The Indigenous Peoples have a much deeper, much more refined understanding, an understanding that the environment and we are so intertwined that if we destroy the environment, we also destroy the human being (P15).

I think that where the Indigenous People are, they preserve the river, the forest, the animals. They learnt through the teachings of what was passed down by their ancestors… I think that if you preserve it, you will reverse this situation that it is today, that nobody knows anymore, nobody is 100% sure of what the climate, the now, the future will be like. tomorrow. (P13)

Today it’s quite complicated for our Indigenous People here in the village, many are no longer planting that swidden they planted before, it’s not worth it anymore, so he’s leaving to work in the city as a mason’s servant, as a day labourer to be able to bring food to his house, for that if you depend on family farming you go hungry. (P14)

… climate change affects in every sense, it interferes physically, psychologically, mentally, culturally, because it is nothing separate, if there was a spring that no longer has drinking water for the Indigenous community, then it will affect the physical, there is no water, there are no forests, there are no animals… (P5).

[…] for example, when there are fires, because of the warming, this harms our territory, our health, our housing… (P7).

The environment, vulnerability and Indigenous mental health were also another analytical group, prevailing the difficulties of cultural maintenance and mental health problems such as suicide. The following quotes represent this analytical category:

Their mental health is also very affected by this lack of opportunity, work, income, support. Drought is a very serious problem here that I have noticed since last year, this year it is very complicated here. So they themselves, due to economic difficulties, also end up having a practice that contradicts their origin, their whole culture, this is very present here. (P14)

[…] it is precisely because of this extreme lack of perspective on life, of motivation to live, that alcohol and drugs finally become an escape from the miserable reality they live. The incidence of alcohol and drugs in the Indigenous community is very high, perhaps the most decimating agent of Indigenous culture. […] Here (village) everything (drugs and drink) comes in. They become addicted, and they have to steal, find a way to get money to buy the drug. We had a recent case of death of a child whose mother was an alcoholic and left the child without food and ended up dying, very dramatic. (P3)

[…] many of the Indigenous People end up going into depression due to living in a culture strongly influenced by whites, losing their ethnic origins, being in an existential limbo that leads to suicide and they also have access to alcohol from a very early age and alcohol makes them also commit crimes, commit suicide…. (P8)

Suicide is a reality […] and among the Indigenous population it is gigantic, […] there is this issue of land, of belonging, of not belonging, of how to deal with the problems, many times they somatize everything and then suicide is the only way that perhaps many of them believe to be the solution… (P1)

Finally, the need to promote public and health policies for Indigenous peoples with Indigenous people involved in the decisions made about them and their lands. This will ensure that the interconnectedness between their communities, nature and health is understood and protected. The following quotes represent this analytical category:

I understand that those who know what is best for the Indigenous People are the Indigenous People themselves, they have enough capacity to decide what is best for them. They have the ability to decide on health, education, social work, basic sanitation, the environment. (P1)

In the first place, it is bringing those populations that are most affected, […] bringing traditional peoples to occupy decision-making positions, we need to do that. Need these policies to be made by those who are affected and think about the direct consequences. (P21)

Demarcation of Indigenous lands is the first point and from the demarcation of Indigenous lands, the development of public policies so that they can remain in these territories and have conditions for this, including public policies, so that they can manifest their language and culture anywhere. (P1)

Before talking about public policy for Indigenous health, the first action must be to guarantee the right to land, with protection of their sacred territories because everything is interconnected. Secondly, the qualification of special Indigenous health teams to act in the prevention and promotion of mental health and network articulation for the treatment of more delicate cases. (P9)

You walk in the village, you walk in the schools, you see dirty people, it’s not because they are filthy, because they like dirt, it’s because of the lack of water, which they don't even have to drink. So what public policies do I recommend? I recommend policies that guarantee human dignity, such as access to water, investments so that each residence can have a water tank, because sometimes there is and there is not enough for a long period. (P18)

If you don't have potable water, ready to drink, you are subject to going to a dam sometimes and getting water that is unfit for consumption. (P12)

**Figure 1:**
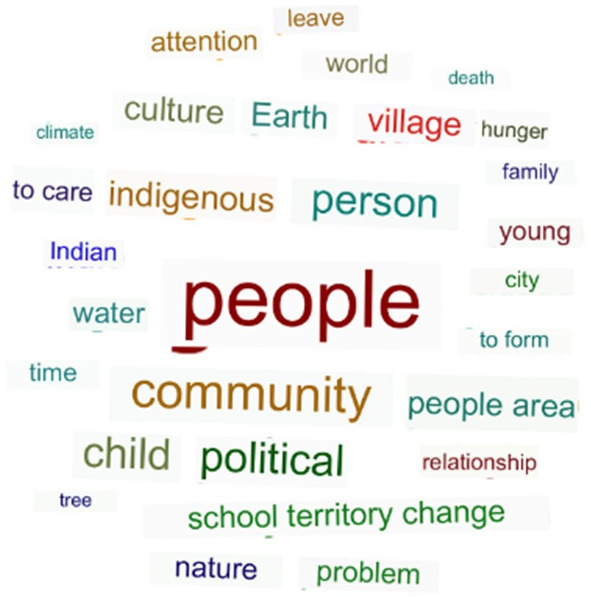
Conceptual representation about climate change and health in the Brazilian Indigenous context.

The extracted concepts obtained by means of the relations between two passages of citations, codes and categories represent a synthesis of the proposed research object, connecting all the categories, subcategories and codes. Figure 1 illustrates the main conceptual representation about climate change and health in the Brazilian Indigenous context.

## Discussion

The perspectives of Indigenous stakeholders and public service managers on the interconnectedness of climate change and Indigenous health were deeply entrenched in their lived experiences of losing their lands from deforestation and environmental degradation and impacts on customs, livelihoods and health of their communities. We highlight some salient points related to the key themes that emerged from the interviews.

### Environmental degradation and climate change

Environmental degradation and climate change, fueled by global warming, have emerged as significant threats to quality of life, as highlighted by participants in this study. Indigenous peoples, recognised as stewards of the Earth, are disproportionately affected due to their close relationship with the environment and reliance on natural resources.

This impact is amplified for those residing in vulnerable areas near rivers, slopes and forests, exacerbating existing inequalities, marginalisation and the enduring legacy of colonisation. Consequently, climate change poses a public health crisis across Latin America and the Caribbean, a region with numerous low-income countries characterised by fragile economies and limited healthcare access. Home to over 40 million Indigenous people whose health and well-being are inextricably linked to the environment, the region faces unique challenges in mitigating the adverse effects of climate change on its most vulnerable populations.^19^

The ongoing process of environmental degradation poses a grave threat to the survival and cultural integrity of Indigenous peoples. The depletion of natural resources essential for their traditional livelihoods, coupled with the potential for escalated conflicts over dwindling resources, could ultimately lead to devastating consequences, including the risk of cultural genocide.^20 21^ Ecosystem conservation is paramount for Indigenous peoples, as their culture, worldview (cosmovision) and survival are intrinsically linked to nature. They not only depend on a balanced environment for their livelihoods but also interpret natural signs as indicators for various events, further emphasising the critical importance of preserving their ecosystems.^10 22^Indigenous subsistence practices have evolved over time, incorporating various customs. Despite these changes, Indigenous peoples maintain a deep understanding of themselves as an integral part of nature. They establish balanced, non-monetary exchange systems that preserve biomes and biodiversity, using natural resources without jeopardising the ecosystem.

Participants emphasised that environmental degradation and climate change disrupted subsistence agriculture, forcing the replacement of traditional Indigenous crops. This shift is problematic because traditional crops, as integral components of the ecosystem, play a crucial role in conserving nature.^23 24^ The traditional knowledge, cultural practices, land-use patterns and resource management systems employed by Indigenous peoples have historically played a crucial role in safeguarding biodiversity, maintaining hydrological cycles, curbing deforestation, preserving forest carbon stocks and providing vital environmental services that contribute to the stability of climatic conditions.^25^ Indigenous lands harbour unique elements that contribute to improved living conditions for society, extending beyond environmental benefits to economic advantages as well. The wealth of traditional knowledge and Indigenous socio-biodiversity offers significant potential for generating income through a variety of products and services. The concept of a bioeconomy that promotes sustainable environmental management while respecting the rights of Indigenous peoples and fostering their own development (ethnodevelopment) presents a promising avenue for addressing the challenges faced by these communities.^26^

The migration of Indigenous peoples to urban centres across Latin America is a growing social phenomenon, driven by a complex network of factors. Among these, the loss of Indigenous territories and environmental destruction play a significant role. It is estimated that more than 200 million people in the world will be forced to leave their regions due to climate change.^27^ This displacement forces individuals and entire communities to abandon their territories, disrupting their livelihoods, altering production systems and jeopardising their very survival.^4^

### Environment, vulnerability and impact on Indigenous mental health

Within the Brazilian context, the concept of mental health among Indigenous populations has been widely debated. Generally, it is understood as encompassing individual, family, social or community well-being, often referred to as ’good living’.^25 28^ Indigenous peoples demonstrate a significantly higher prevalence of mental distress compared with majority populations. This heightened vulnerability is influenced by a complex interplay of historical traumas, including the enduring legacy of colonialism, racism, slavery and land dispossession.^29 30^ Brazilian Indigenous peoples are experiencing a dramatic escalation of land grabbing, illegal logging, mining, invasions and even the establishment of unauthorised settlements within their traditional territories. This escalating conflict over Indigenous lands has reached alarming levels, threatening the very existence of numerous Indigenous communities in Brazil. The resulting social and environmental upheaval has severe repercussions on mental health, exacerbated by factors such as social marginalisation, disrupted lifestyles and livelihoods and exposure to violence.^31 32^

The report ‘Violence Against Indigenous Peoples in Brazil’ reveals a concerning rise in documented violence and the highest number of Indigenous suicides in recent years. The loss of territory and agricultural land, racism, poverty, social vulnerability and inadequate healthcare access have been identified as key contributing factors to this alarming increase in suicides.^7^ The proliferation of alcohol and other drugs within numerous Indigenous villages is a growing concern. While attributed to various factors, this issue is particularly linked to the limited opportunities and the lack of prospects faced by Indigenous peoples.

The Brazilian Ministry of Health recognises the Indigenous population as vulnerable and experiencing a high incidence of psychosocial issues, including chemical dependency (alcohol and other drugs), misuse of psychotropic medications, suicide and violence. This alarming situation is attributed to the disruption of traditional ways of life, challenges in securing economic subsistence and exposure to conflicts. These factors contribute to significant suffering within Indigenous communities, often leading to self-destructive behaviours.^7 32^ The alarming deterioration of mental health among Indigenous peoples in Brazil, tragically culminating in a disproportionately high suicide rate, underscores the heightened vulnerability experienced by this marginalised population. This stark disparity reflects the systemic denial of fundamental rights and the inadequacy of existing public policies. The suicide mortality rate among Indigenous individuals in Brazil, at 15.2 per 100 000, is nearly three times that observed in the non-Indigenous population (5.7 per 100 000), highlighting the urgent need for targeted interventions and culturally sensitive mental health support.^4^

### Public health actions and policies for Indigenous peoples

Promoting Indigenous health necessitates a multi-sectoral collaboration that prioritises Indigenous voices and perspectives. Such collaborative efforts enable the implementation of integrated actions across various levels of government and society, ultimately striving to improve the quality of life and promote health and well-being for Indigenous communities. This need for a comprehensive and interconnected approach was a recurring theme in participant discussions regarding public health policies, with many emphasising the fundamental importance of guaranteeing the right to Indigenous lands as a non-negotiable prerequisite for their health and well-being. One of the most contentious issues in Brazil revolves around the demarcation of Indigenous lands, defined as areas inhabited by Indigenous peoples for their productive activities, cultural preservation and the continuation of their traditions. An Indigenous land is not merely a physical space; it is a fundamental component of cultural and religious identity, ensuring the survival of Indigenous communities and serving as their ancestral territory.^29 33^ Indigenous peoples maintain a harmonious relationship with their cultures and spirituality, actively engaging in the protection, preservation and continued development of their traditions to ensure the transmission and flourishing of their collective identity for future generations.^4^

Water scarcity has emerged as a pressing global concern. Indigenous communities, acutely aware of the consequences, actively seek to conserve uncontaminated springs and restore those that have been polluted or damaged. This proactive approach stems from the understanding that environmental degradation and water scarcity directly threaten their survival. Beyond land and water rights, participants highlighted the need for comprehensive public policies and government actions that improve the quality of life for Indigenous people across multiple domains, including access to healthcare, education, sports, leisure, culture and infrastructure. They emphasised the importance of stricter legislation to prevent mining and deforestation on Indigenous lands.

### Strengths and limitations of the study

This study embraced a culturally sensitive approach, building trust and rapport with Indigenous participants. This grounding in the Brazilian context enhances the relevance and applicability of the findings.

Despite these strengths, the study has limitations. Expanding the sample size and diversity to include a broader range of ethnicities, indigenous policymakers and young people from various villages would have enriched the study’s interpretive value and ensured a wider representation of Indigenous perspectives.

Furthermore, acknowledging the diverse cultural practices and beliefs within different Indigenous communities is crucial. While the restoration of communal balance, harmony and collective well-being is a shared concern, the specific challenges stemming from environmental degradation vary across ethnic groups due to distinct historical, geospatial and cultural contexts.

### Conclusion

Indigenous communities are disproportionately affected by environmental degradation and climate change, further compounded by challenges related to the demarcation of Indigenous lands, essential for their survival and cultural preservation. To address these pressing issues, they argue strongly that it is imperative to implement and strengthen intersectoral actions that prioritise environmental preservation, including water conservation, forest protection and sustainable agricultural practices, thereby ensuring a brighter future for these communities. Traditional practices and knowledge foster a sense of belonging and cultural rootedness, but the lack of access to essential resources and the challenges of integration into mainstream society can create vulnerabilities. Furthermore, a complex interplay exists between the environment and Indigenous mental health, often associated with substance abuse and contributing to alarmingly high suicide rates. Public health policies need to be developed with Indigenous peoples to address these multifaceted challenges. A holistic approach to health, coupled with collaborative strategies that value the strengthening of connections with nature and community, are fundamental to promote the overall well-being of Indigenous peoples.

## supplementary material

10.1136/bmjopen-2023-083624 online supplemental file 1

## Data Availability

Data are available upon reasonable request.
