# Supplementary material for: Environmental degradation, climate change and health from the perspective of Brazilian Indigenous stakeholders: a qualitative study
Source: BMJ Open. 2024 Sep 24;14(9):e083624. doi: 10.1136/bmjopen-2023-083624 (PMC11429271; doi:10.1136/bmjopen-2023-083624)
Supplement: online supplemental file 1 [file bmjopen-14-9-s001.pdf]

Interview Script

Vignette 1

An indigenous Brazilian activist took part in the official opening of the Climate Summit Conference (COP26) in Glasgow, Scotland in 2021. In her speech, the 24-year-old spoke about climate change and indigenous peoples. I'm going to show an excerpt from this speech:

Show video - Vignette 1 (https://bit.ly/3SwZsmq)

Questions

1 - What did you think of the video? Comment on the subject

2 - What caught your attention in the video? Why?

3 - In your opinion, could climate change affect the lives of indigenous people? How? In what way?

4- Could you comment further on public policies and climate change in Brazil? Can you name some actions that could help improve existing problems?

Vignette 2

Mental health problems are common among people all over the world, and among the indigenous population too. The following video illustrates some of this problem

Show video - Vignette 2 (https://bit.ly/3A6g6Tt)

1 - What did you think of the video?

2 - Which part caught your attention the most?

3 - Do you think climate change could affect the physical or mental health of indigenous people? Why? In what way? How?

4 - How could the indigenous person's death have been avoided?

5 - Would you like to comment further on public policies related to the mental health of indigenous people in Brazil? Can you name some actions that could help improve the existing problems?
