# Supplementary material for: Environmental degradation, climate change and health from the perspective of Brazilian Indigenous stakeholders: a qualitative study
Source: BMJ Open. 2024 Sep 24;14(9):e083624. doi: 10.1136/bmjopen-2023-083624 (PMC11429271; doi:10.1136/bmjopen-2023-083624)
Supplement: Supplementary file 2 [file bmjopen-2023-083624.reviewer_comments.pdf]

## PEER REVIEW HISTORY

BMJ Open publishes all reviews undertaken for accepted manuscripts. Reviewers are asked to complete a checklist review form and are provided with free text boxes to elaborate on their assessment. These free text comments are reproduced below.

## ARTICLE DETAILS

| | |
|---|---|
| **TITLE (PROVISIONAL)** | Environmental degradation, climate change and health from the perspective of Brazilian Indigenous stakeholders: A qualitative study |
| **AUTHORS** | Grande, Antonio José; Dias, Ieda M.A.V.; Jardim, Paulo T. C.; Aparecida Vieira Machado, Alessandra; Soratto, Jacks; da Rosa, Maria Inês; Ceretta, Luciane Bisognin; Zourntos, Xanthi; Suares, Regeane Oliveira; Harding, Seeromanie |

## VERSION 1 - REVIEW

| | |
|---|---|
| **REVIEWER NAME** | Brubacher, Laura |
| **REVIEWER AFFILIATION** | University of Waterloo, School of Public Health Sciences |
| **REVIEWER CONFLICT OF INTEREST** | None. |
| **DATE REVIEW RETURNED** | 05-Mar-2024 |

| | |
|---|---|
| **GENERAL COMMENTS** | Environmental degradation, climate change and health from the perspective of Brazilian Indigenous stakeholders: Qualitative interview study<br><br>Introduction:<br>• I would recommend a re-structuring of this introduction. The first four paragraphs are very short and could easily be more streamlined/merged. As written, it takes until the fifth paragraph to even mention environmental changes, despite that being the central focus of this study as I understand it. At most, start with one paragraph that provides broader context on the health of Indigenous Peoples in Brazil. Then, be sure to move to discussing environmental changes and their potential impact on health – this is the important background for understanding your study's results that follow.<br>• Additionally, the background you provide on climate change and health, particularly the health of Indigenous Peoples, needs to be more robust. There is a very rich literature at this climate-health interface which needs to be more effectively engaged with to position your study among the broader scholarship.<br><br>Methods:<br>• Study type, first paragraph: This is a very long and unclear sentence. Please re-write for clarity. Also, please provide more clarity, specificity, and detail as to how you are using political ecology as a theoretical lens in this study. Was it used to shape the data collection tools (i.e., interview guide) – if so, how? Was this a hybrid inductive-deductive analysis? Did the political ecology theoretical lens then inform your analysis – if so, how?<br>• Procedures for selecting participants: More concision required – I would recommend streamlining into one tight paragraph.<br>• Data collection: I am intrigued by your use of vignettes and interested in the methodology. However, it is currently very unclear |

| | |
|---|---|
| | how the vignettes were integrated into your semi-structured interview, and the overall data collection approach. More specifics are needed (perhaps an interview guide as supplementary material as well). Additionally, more information is needed as to your rationale for using vignettes – how did this enhance your understanding of the research topic, deepen your inquiry, etc. What does this methodology afford that cannot be afforded by a more ‘traditional’ semi-structured interview?<br>• Box 1: Not necessary. The associated text that provides details around your use of the G-suite package is also not necessary to include in a manuscript.<br>• Data analysis: This currently reads as from a textbook (very generic steps associated with thematic content analysis) – can you be more specific and anchor this to your process more clearly?<br>• I would highly recommend a paragraph (up front in your methods) that positions the research team. Who is part of this team (your positionality – Indigenous, non-Indigenous)? Does the work emerge from existing community priorities, or existing partnership among the research team?<br><br>Results and Discussion:<br>• It appears that you have created a combined results/discussion section. Participant quotes are currently integrated into your discussion. These are your results so should be moved into the results section. As a qualitative study, these are your findings. I am far less interested in the proportion of quotes related to a particular code – your current results – as I am in a rich, descriptive synthesis of your participant quotes.<br>• Please discuss the strengths and limitations of your study.<br><br>Other:<br>• Please capitalize Indigenous Peoples throughout your manuscript. This is now common and expected within the Indigenous health scholarship.<br>• Please conduct a thorough copy-edit, as there are currently many grammar and other editorial errors throughout the manuscript (unclear or incomplete sentences, etc.).<br>• I am uncertain of the journal’s requirements for reference formatting and, in particular, for language. Many of the references are cited in Portuguese, I am assuming (I have no proficiency). I am unclear whether this is how they were formatted using your reference management software, or if they are actually only available in another language than English. It makes it difficult for me to assess the literature that was engaged with and whether or not it was appropriate to this study.<br><br>Thank you for the opportunity to review your manuscript. I hope my feedback is useful and constructive to you. |

| | |
|---|---|
| **REVIEWER NAME** | Hill , Kyle |
| **REVIEWER AFFILIATION** | University of Minnesota |
| **REVIEWER CONFLICT OF INTEREST** | None |
| **DATE REVIEW RETURNED** | 09-Apr-2024 |

| | |
|---|---|
| **GENERAL COMMENTS** | This is an important contribution to the literature on climate impacts to the health and welfare of Indigenous communities in Brazil. My |

| | |
|---|---|
| | recommendation to authors is a minor revision for publication. I am sensitive that this review considers that language differences that may exist between authors and the use of English in the manuscript. I will detail some considerations for revisions:<br><br>1. The Introduction and throughout the paper "The Indigenous People" does not adequately situate the manuscript relative to the communities who are participating. Please identify the country of origin, even if the particular Tribe or community does not want to be identified. Indigenous People is too broad.<br>2. Later in the abstract introduction is mentioned "two Indigenous Peoples", again, this needs to be revised to include communities, by country or whether this does, in fact, refer to two (2) Indigenous people or individuals.<br>3. "this group" in the introduction might be referring to this generation of youth, consider revising this term.<br>4. Introduction on page 4 - "late care" consider revising to "disparities in access or unavailability of care".<br>5. Under page 7, Data collection, please revise the "Indigenous People and their important role in maintaining climate change" - This sentence does not represent the general themes of Indigenous activists at COP26. "Maintaining climate change" is what Indigenous peoples are protecting against, organizing against and defending against. I wonder if we can consider whether this phrase does not represent how Indigenous communities' traditional practices are helping to mitigate or adapt to climate change?<br>6. It is unclear how the vignettes are important for this investigation.<br>7. Please elaborate on how Google drive and google suite was encrypted or somehow able to ethically store data, e.g., is it encrypted?<br>8. Revision needed here on page 16 line 55 - the citation requires parentheses.<br>9. Please add limitations section in the discussion or another section. |

**VERSION 1 – AUTHOR RESPONSE**

Reviewer: 1

Dr. Laura  Brubacher, University of Waterloo

Comments to the Author:

See attached.

Reviewer: 2

Dr. Kyle   Hill , University of Minnesota

Comments to the Author:

This is an important contribution to the literature on climate impacts to the health and welfare of Indigenous communities in Brazil. My recommendation to authors is a minor revision for publication. I

am sensitive that this review considers that language differences that may exist between authors and the use of English in the manuscript. I will detail some considerations for revisions:

1. The Introduction and throughout the paper "The Indigenous People" does not adequately situate the manuscript relative to the communities who are participating. Please identify the country of origin, even if the particular Tribe or community does not want to be identified. Indigenous People is too broad.

Thank you for your time and expertise in reviewing our work. Your valuable recommendations significantly improved our paper.

We have added the paragraph

The Indigenous participants in this study have faced challenges in maintaining their demarcated lands and preserving their deep spiritual and mythical connection to nature. Storytelling remains a vibrant tradition, reinforcing their identity and sense of belonging. School lunches incorporate traditional foods, and the curriculum emphasizes oral learning methods and participation in village activities alongside community leaders, shamans, parents, and elders.

2. Later in the abstract introduction is mentioned "two Indigenous Peoples", again, this needs to be revised to include communities, by country or whether this does, in fact, refer to two (2) Indigenous people or individuals.

Thank you.

We made corrections, there was a typo.

The Indigenous participants in this study have faced challenges in maintaining their demarcated lands and preserving their deep spiritual and mythical connection to nature. Storytelling remains a vibrant tradition, reinforcing their identity and sense of belonging. School lunches incorporate traditional foods, and the curriculum emphasizes oral learning methods and participation in village activities alongside community leaders, shamans, parents, and elders.

3. "this group" in the introduction might be referring to this generation of youth, consider revising this term.

The whole paragraph was restructured

Non-communicable chronic diseases, such as obesity, arterial hypertension, type II diabetes, and psychiatric disorders, significantly impact Indigenous health. The rising prevalence of these conditions in Indigenous children is closely tied to changes in subsistence strategies, dietary habits, and physical activity levels, resulting from complex sociocultural and economic transitions.

4. Introduction on page 4 - "late care" consider revising to "disparities in access or unavailability of care".

Thank you

We accepted it.

5. Under page 7, Data collection, please revise the "Indigenous People and their important role in maintaining climate change" - This sentence does not represent the general themes of Indigenous activists at COP26. "Maintaining climate change"  is what Indigenous peoples are protecting against, organizing against and defending against. I wonder if we can consider whether this phrase does not represent how Indigenous communities' traditional practices are helping to mitigate or adapt to climate change?

I understand. To better align with your goals, I've refined the statement to emphasize the connection between your findings and the broader issue

We corrected it to

Indigenous peoples' deep connection to their lands and traditional practices plays a crucial role in both mitigating and adapting to climate change. Their ecological knowledge and sustainable resource management strategies offer valuable insights for environmental conservation and resilience in the face of a changing climate.6. It is unclear how the vignettes are important for this investigation.

Vignettes, as brief and carefully crafted representations of individuals or social environments, have proven effective in exploring sensitive topics and eliciting in-depth responses and reflexivity from participants. By accurately referencing key elements, vignettes prompt interviewees to make decisions or form judgments, providing valuable insights into their perspectives and thought processes.

7. Please elaborate on how Google drive and google suite was encrypted or somehow able to ethically store data, e.g., is it encrypted?

Google prioritizes data security by employing two key encryption methods:

Encryption at rest: Data stored on disks or backup media is encrypted, rendering it unreadable to unauthorized individuals even with physical access to the storage equipment.

Encryption in transit: Data transmitted over the internet or within Google's network is encrypted, ensuring its protection during transmission between data centers.

This dual approach safeguards user data both when it's stored and when it's being moved, providing a robust defense against potential security breaches.”

8. Revision needed here on page 16 line 55 - the citation requires parentheses.

Thank you

9. Please add limitations section in the discussion or another section.

A more extensive and diverse sample encompassing a wider range of ethnicities, indigenous policymakers from various villages, and young people would have enhanced the study's interpretive value. Prioritizing voice and representation is crucial for developing sustainable interventions and achieving system-wide change.

Reviewer 2

bmjopen-2023-083624 – Reviewer Comments to Authors

Environmental degradation, climate change and health from the perspective of

Brazilian Indigenous stakeholders: Qualitative interview study

Introduction:

• I would recommend a re-structuring of this introduction. The first four paragraphs are very short and could easily be more streamlined/merged. As written, it takes until the fifth paragraph to even mention environmental changes, despite that being the central focus of this study as I understand it. At most, start with one paragraph that provides broader context on the health of Indigenous Peoples in Brazil. Then, be sure to move to discussing environmental changes and their potential impact on health – this is the important background for understanding your study's results that follow.

• Additionally, the background you provide on climate change and health, particularly the health of Indigenous Peoples, needs to be more robust. There is a very rich literature at this climate-health interface which needs to be more effectively engaged with to position your study among the broader scholarship.

We have restructured the introduction

Brazil's Indigenous population, now over 1.65 million according to the 2022 Census, has seen a notable increase due to higher birth rates compared to the non-Indigenous population. However, this growth is accompanied by significant health challenges. Indigenous communities often face precarious sanitation conditions, leading to intestinal infections, while hepatitis and malaria remain major causes of morbidity and mortality.

Additionally, non-communicable chronic diseases like obesity, hypertension, type II diabetes, and psychiatric disorders are on the rise, particularly among children. These trends are linked to shifts in subsistence, diet, and physical activity resulting from complex sociocultural and economic transformations.

While cancer incidence is low, mortality rates remain high due to disparities in healthcare access. Violence also contributes significantly to mortality rates.

Environmental changes pose another critical threat to Indigenous health. Ecosystem degradation, rising temperatures, and extreme weather events are major concerns, with the World Health Organization recognizing climate change as the greatest challenge to global health. Indigenous peoples, deeply connected to nature, are particularly vulnerable to these changes. Recent reports of mercury contamination in Amazonian Indigenous communities highlight this vulnerability.

The Indigenous participants in this study, striving to maintain their demarcated lands and spiritual connection to nature, possess a rich storytelling tradition that reinforces their identity and belonging. Their schools integrate cultural foods and oral learning methods, emphasizing participation in village activities with community leaders, shamans, parents, and elders.

This study aims to analyze the understanding of Indigenous stakeholders and public managers regarding the interconnectedness of climate change and Indigenous health.

Methods:

• Study type, first paragraph: This is a very long and unclear sentence. Please re-write for clarity. Also, please provide more clarity, specificity, and detail as to how you are using political ecology as a theoretical lens in this study. Was it used to shape the data collection tools (i.e., interview guide) – if so, how? Was this a hybrid inductive-deductive analysis? Did the political ecology theoretical lens then inform your analysis – if so, how?

Adjusted

• Procedures for selecting participants: More concision required – I would recommend streamlining into one tight paragraph.

The study included 22 Indigenous stakeholders and public managers. Inclusion criteria encompassed:

a) Managers: Individuals over 18 years old, occupying management positions at municipal, state, or federal levels within the executive, legislative, or judicial branches, and working in Indigenous areas.

b) Indigenous: Individuals over 18 years old, self-identifying and recognized as belonging to an ethnic group with distinct cultural characteristics from the broader national society.

• Data collection: I am intrigued by your use of vignettes and interested in the

methodology. However, it is currently very unclear how the vignettes were integrated into your semi-structured interview, and the overall data collection approach. More specifics are needed (perhaps an interview guide as supplementary material as well). Additionally, more information is needed as to your rationale for using vignettes – how did this enhance your understanding of the research topic, deepen your inquiry, etc. What does this methodology afford that cannot be afforded by a more 'traditional' semi-structured interview?

Evidence suggests that vignettes are an effective method for exploring sensitive or difficult topics, encouraging in-depth responses and reflexivity from participants.

The interviews incorporated vignettes, beginning with profile questions on gender, age, experience in Indigenous health/management, and additional employment. Two vignettes were then presented, followed by associated questions to elicit participant responses and reflections.

• Box 1: Not necessary. The associated text that provides details around your use of the G-suite package is also not necessary to include in a manuscript.

We kindly disagree due to the data security clarity.

Google prioritizes data security through robust encryption measures:

1. Encryption at rest: Data stored on disks or backup media is encrypted, ensuring it remains unreadable even if someone gains unauthorized physical access to the storage equipment.

2. Encryption in transit: Data transmitted over the internet or within Google's network is encrypted, protecting it as it moves between data centers.

This comprehensive approach safeguards user data throughout its lifecycle, offering strong protection against potential security breaches.

• Data analysis: This currently reads as from a textbook (very generic steps associated with thematic content analysis) – can you be more specific and anchor this to your process more clearly?

Thank you. We have adjusted in the text.

• I would highly recommend a paragraph (up front in your methods) that positions the research team. Who is part of this team (your positionality – Indigenous, non-Indigenous)? Does the work emerge from existing community priorities, or existing partnership among the research team?

Our long-established Indigenous community-academic partnerships recognise it is vital to respect cultural integrity and agency through the integration of Indigenous knowledge and values. This research question emerged as we have been working in partnership with them since 2017.

Results and Discussion:

• It appears that you have created a combined results/discussion section. Participant quotes are currently integrated into your discussion. These are your results so should be moved into the results section. As a qualitative study, these are your findings. I am far less interested in the proportion of quotes related to a particular code – your current results – as I am in a rich, descriptive synthesis of your participant quotes.

• Please discuss the strengths and limitations of your study.

Due to journal formatting constraints, we had to adjust the structure of the text.

Study limitations

The study's interpretive value could be enhanced by incorporating a larger and more diverse sample, including a wider range of ethnicities, indigenous policymakers from various villages, and young people. Prioritizing diverse voices and representation is crucial for developing effective, sustainable interventions and fostering system-wide change.

Other:

• Please capitalize Indigenous Peoples throughout your manuscript. This is now common and expected within the Indigenous health scholarship.

Thank you

• Please conduct a thorough copy-edit, as there are currently many grammar and other

editorial errors throughout the manuscript (unclear or incomplete sentences, etc.).

A native English speaker has reviewed and revised the text.

• I am uncertain of the journal's requirements for reference formatting and, in particular, for language. Many of the references are cited in Portuguese, I am assuming (I have no

proficiency). I am unclear whether this is how they were formatted using your reference

management software, or if they are actually only available in another language than

English. It makes it difficult for me to assess the literature that was engaged with and

whether or not it was appropriate to this study.

A native English speaker has reviewed and revised the text.

Thank you for the opportunity to review your manuscript. I hope my feedback is useful and constructive to you.
